# Inclusivity and decolonisation of the post-graduate public health curriculum: Reflections from a student-led approach

**DOI:** 10.1016/j.puhip.2024.100507

**Published:** 2024-05-17

**Authors:** G. Carleton-Boylan, S. Crossley, P. Siebert, N. Ajanaku, A. Iqbal, A. John, S. Sandhu, C. Williams, L. Leach, R. Patel, H. Buchanan, J. Taggar, J. Leonardi-Bee, J. Morling, I. Qureshi, L. Hubber, J. Bethea, E.E. Wilson

**Affiliations:** a Public Health, Deputy Director of Public Health Postgraduate Programmes, Director of PGT Quality Assurance, Nottingham Centre for Public Health and Epidemiology, University of Nottingham, United Kingdom; b Public Health, Lead for Student Experience, Nottingham Centre for Public Health and Epidemiology, University of Nottingham, United Kingdom; c Public Health, Director of Public Health Postgraduate Programme, Nottingham Trent University, United Kingdom; d Global Health Student, University of Nottingham, United Kingdom; e Anatomy and Vascular Biology, Faculty of Medicine and Health Sciences BAME Champion, School of Health Sciences, University of Nottingham, United Kingdom; f Medical Education, Dean of Medicine, BAME Champion, Queen Mary University, London, United Kingdom; g Health Psychology, Academic Unit Director for Postgraduate Education, Nottingham Centre for Public Health and Epidemiology, University of Nottingham, United Kingdom; h Primary Care Education, Academic Unit Director for Undergraduate Education, Nottingham Centre for Public Health and Epidemiology, University of Nottingham, United Kingdom; i Medical Statistics and Epidemiology, Director of Nottingham Centre for Evidence Based Healthcare, Nottingham Centre for Public Health and Epidemiology, University of Nottingham, United Kingdom; j Public Health, Deputy Director of Academic Unit, Lead for Clinical Academic Public Health Training, Nottingham Centre for Public Health and Epidemiology, University of Nottingham, United Kingdom; k Global Health, Nottingham Centre for Public Health and Epidemiology, United Kingdom; l Public Health, Nottingham City Council, Nottingham Centre for Public Health and Epidemiology, University of Nottingham, United Kingdom; m Public Health, Nottinghamshire Healthcare Trust, Academic Public Health Training Director East Midlands, Health Education England, Nottingham Centre for Public Health and Epidemiology, University of Nottingham, United Kingdom; n Director of Public Health Postgraduate Programmes, Faculty Lead for Educational Excellence, Nottingham Centre for Public Health and Epidemiology, University of Nottingham, United Kingdom

## Abstract

The future of successful public health practice requires public health students to be educated within a decolonised curriculum that challenges the historical biases and inequalities that are deeply embedded within global public health and society. In this commentary, we reflect on what it can mean and why it's important to decolonise and diversify a public health curriculum. We describe how we used a student-led approach to begin this process, and share recommendations that are applicable to national and international curricula.

## 1 Introduction

Decolonisation, in its broadest sense, involves identifying the colonial systems, structures and relationships that exist, exploring how they have shaped our modern world, and working to challenge and remove these structures entrenched within society [1]. With regard to public and global health, decolonisation is not a historic exercise. While formal colonial structures have largely been removed, significant global inequalities remain embedded within public and global health structures, where the health problems, healthcare structures and the available resources to target public health issues of high-income countries are often very different to those of low-middle income countries (LMICs) [2,3].

This is equally true with the allocation of research funding within the global public health field [4], meaning that the research which is viewed as “higher quality” on the global stage largely comes from high-income countries, and in Universities, there is a drive to deliver research-led teaching which predominately showcases “high-impact” research [5]. This perpetuates the cycle of inequity as LMIC research receives less academic exposure, impacting future research prioritisation and investment [4]. All of this is indicative of a Western, Educated, Industrialized, Rich and Democratic (WEIRD) research perspective which can fail to consider the psychological and material differences amongst global populations [6].

Decolonisation of the postgraduate public health curriculum, therefore, seeks to educate future public and global health practitioners with a truly global perspective that reflects the historical biases and inequalities that are deeply embedded within global public health and society [6]. Decolonisation of the curriculum also aims to challenge the entrenched power systems that can impact decision-making for best pedagogical practice, and the wider structural, institutional and educational barriers that underpin educational experience [7,8].

In this commentary, we reflect on what it can mean and why it's important to decolonise a public health curriculum, describe how we used a student-led approach to begin this process, and share findings and recommendations that are applicable to national and international public health curricula. The aim of this commentary is to open a wider discussion around how best to encourage students to become the central driving force in postgraduate curriculum design and development and to stimulate continuous dialogue and review of curricula, working towards achieving a fully inclusive and decolonised curriculum [7].

## 2 Why did we decide to start decolonising and diversifying our public health curriculum?

Student feedback identified that not all students were not all having equivalent education experiences on the course – a finding often reported in the literature. Students from ethnic minority backgrounds can have poorer experiences and outcomes of higher education. This is due to multiple factors, including discriminatory teaching, learning and assessment practices, subtle exclusion and segregation, lower teacher expectations, prejudiced attitudes and inadequate student support mechanisms [9,10]. In the UK, there is a 16.1 % attainment gap between the number of high-quality degrees (1st or 2:1) awarded to White UK-domiciled students compared to UK-domiciled students from ethnic minority groups, and a 15 % attainment gap with non-European Union students [[11], [12], [13]] [[11], [12], [13]] [[11], [12], [13]]. Given our changing student demographic, with 35.9 % of our students being UK residents, and 64.1 % of our students being International, we were further motivated to create an inclusive environment for all students, where all aspects of academia, student life and experience are reviewed in terms of equality and decolonisation.

This project enabled us to present a postgraduate public health and global health curriculum which has a more scientific and historically complete perspective given that some of the very best examples of grassroots, community-led global health advancements are seen in non-Western countries [2], led by relevant global voices [14].

## 3 The importance of our student-led approach

The message from our students was clear: decolonising and diversifying the curriculum was something that we needed to do, and something that they strongly wanted to be involved with. As demonstrated by the National Union for Students’ “Race for Equality” report [13], a student-led approach is crucial when developing curricular and higher-education experience to ensure that any developments are specific to the needs and experiences of students. Based on constructivist learning theory, co-creation of teaching and learning resources moves students from passive to active participants in their learning journeys, enriching the learning environment for all through the production of knowledge which is truly reflective of the experience and needs of the students [15].

## 4 What we have done to start decolonising and diversifying our curriculum

In 2020, we started to collect student feedback on areas of the curriculum which needed to be diversified. Initially, this was largely from a topic perspective rather than an inclusivity and decolonisation perspective. However, through this exercise, it became apparent that the topic gaps students identified in the curriculum provision tended to align with international and emerging societal and public health perspectives.

In 2021, a group of 10 postgraduate public health and global health students led a full curriculum review exploring the equality, diversity, inclusivity and decolonisation of the postgraduate public health courses at the University of Nottingham. The content, assignment structure and teaching methods of every module, as well as wider student support and experience were independently reviewed in their entirety by three students, and findings were triangulated through discussion. As the students involved were a mix of home and international students and were diverse in terms of demographics and academic background, students viewed content from different perspectives, ensuring that review findings would be representative of wider viewpoints.

We communicated with students and staff through a number of different channels including weekly public health team meetings, student feedback sessions and student representative meetings. Additionally, there were meetings with the Faculty of Medicine and Health Sciences Black and Minority Ethnic (BAME) Champions to further advance the recommendations. There were also feedback meetings with the external examiner for the programmes to assess recommendations in line with academic standards and quality assurance. Additionally, feedback on the recommendations was collated from alumni through social media networks.

We have also shared our review recommendations with two global health external partners, our educational collaborators at neighbouring Nottingham Trent University, and accrediting partners at APHEA and WHO.

## 5 Findings and reflections

Through this project, findings and recommendations around our teaching and learning resources, teaching delivery, assessments, and student support were identified that need to be addressed to create a fully decolonised and diversified curriculum.

While the findings and recommendations presented may be applicable to national and international postgraduate public health (and other) curricula, it is important to note that each curriculum will have areas needing diversification and decolonisation specific to that course. We encourage other courses to look at their specific material, the way in which material is taught, the support available to students, assessment formats, and the environment of the course.

### 5.1 Resources

• *Reading lists were found to be highly Eurocentric*, except for a few resources from the USA. Reading lists should aim to provide varying racial, disability, cultural and gender perspectives. Research from LMICs should also be included in reading lists to help students better understand the realities of LMIC lived experience and expose UK-home students to new perspectives.
• Lecturers should *acknowledge the backgrounds and demographics of students* on the course, and tailor learning materials to reflect these backgrounds. For example, there should be adequate public health examples from LMICs and from the Global South. Lecturers should consider public health practice and policy within different countries from the Global North and Global South, to ensure that students are fully prepared when going into work, and to avoid disadvantaging international students.
• *Presentations should be inclusive* in the following areas: age, disability, race, marriage and civil partnership, pregnancy and maternity, gender reassignment, religion or belief, sex, and sexual orientation. By including images of a diverse range of individuals in presentations, students will feel more represented and included.
• Lecturers should *include the perspectives and experiences of underrepresented populations and minority groups when discussing public health issues.* Including material on how diseases, health issues and lifestyle factors affect groups of individuals differently. Consider the contextual barriers faced by marginalised groups that prevent them from accessing and using health services, engaging in health schemes and abiding by policies.
• When teaching research methodologies in public health, ensure that a *diverse range of evidence synthesis skills* that are widely applicable to a more diverse global public health cohort are taught, and provide examples of how different research methodologies can be applied in local community and global settings.
• When discussing research design, include material on how *some patient groups are typically underrepresented in research* because their needs haven't been met in terms of recruitment and involvement, not necessarily because they don't want to be involved. Help students better understand the barriers to research participation. When discussing intervention design, ensure that populations and communities are not referred to as “hard-to-reach.” Rather, use the term “underserved” as it more accurately considers their lack of inclusion due to various socio-economic, cultural, political, and religious factors that make engagement in research and accessing/using services more difficult for these populations.

### 5.2 Delivery

• There should be a *strong representation of global public health perspectives* regarding the delivery of key public health topics. For example, speakers with lived experience of public health programme delivery within low-income country settings should be invited to teach about public health practice in low-income countries. This may include external expert lecturers with public health practice experience in low-income settings, alumni who have worked in low-income settings, individuals/stakeholders who have experienced health programme delivery in low-income settings, or lecturers from LMICs who would be able to better present the public health problems faced by LMICs [16].
• There should be more *focus on student-led teaching* to maximise the experiences and perspectives of the diverse student cohort. It was felt that this style could also help students navigate the transition between different educational systems and encourage greater participation with critical thinking.
• Some of the teaching sessions in the Public Health curriculum can include *sensitive and hard-hitting topics*, such as COVID-19, sexually transmitted infections and tobacco control, amongst others. Lecturers should approach these topics with care and provide links to resources and services/helplines to potentially sensitive topics, as well as content warnings at the beginning of sessions. It is important to acknowledge that there are also different socio-cultural norms around different health behaviours, such as tobacco use.

### 5.3 Assessment

• Using different forms of assessment, such as presentations, coursework and examinations, will accommodate a wider range of students' academic styles and abilities and improve engagement in the learning process. The use of assessments which have ‘real world’ value such as the production of media resources like public facing podcasts, which allow students to develop and use skills suited to a wide range of learning styles and experience not just those favoured by the dominant orthodoxy in the UK higher education environment and build a portfolio of resources which enhance graduate employability.
• It was found that many *international students struggled with the UK assessment styles*, as they did not align with their previous experiences of academia. An optional class/resource should be introduced to prepare international students for the differences they may experience in UK assessment and learning styles at University. For example, providing information on how to find gaps in the literature, writing academic essays and reports, the importance of referencing correctly, how to handle the pressure of having one assignment determining your grade for a module, and the expectations of UK postgraduate study.
• Having *clear assessment requirements and marking criteria* across all modules would help to eliminate confusion and ambiguity for students, ensuring that all students are evaluated equally and eliminating the possibility of subjective interpretation throughout the assessment process.

### 5.4 Student support

• *Career support* should be provided that is also accessible to international students, who tend to have more difficulty in finding careers after the course due to visa constraints. This highlights an important link between wider educational learning and the role of the wider University setting within the course-level education provision.
• It is important to *consider the unique needs and barriers faced by international students* on post-graduate courses and provide tailored support to reflect these needs. Personal tutors should be assigned to international students at an earlier, pre-induction stage to provide a first point of contact in case of issues with arriving to the UK, such as visa or housing queries. International students have also commented that they struggle with adapting to UK life, finding housing, and not having a ‘sense of belonging’ when coming to University. In response to this, we are co-creating a toolkit with students, including guides on using the UK health system, accessing available mental health and wellbeing services, and finding University accommodation, tips and advice from past UK-home and international students, and information on local public transport, student fees and payments, welfare services, faith support, living in the UK and English language support.
• It is important to *provide tailored support to students from ethnic and cultural minority groups,* as they may face more barriers at University. This should include the organisation of designated staff members who can be responsible as a first point of contact for any students having problems linked with minority groups, acting as a safe space for these students to discuss issues.

### 5.5 Staff-identified barriers to decolonisation

Throughout the review process, academic staff provided feedback and insight into the discussions. Staff were fully supportive of the need to develop a fully decolonised and inclusive curriculum but identified a number of important barriers which need to be addressed at the institutional level in order to ensure inclusive and decolonisation curriculum development processes are constant throughout the academic cycle.

• *Training and development:* Staff identified that they often are not fully conversant and up-to-date with all of the rapidly evolving aspects of EDI and decolonisation, and regular updating training needs to be provided in order to ensure they can update teaching materials confidently.
• *Resources:* Staff workloads in higher education institutions are high, and in order to continually update teaching materials sufficient resource needs to be provided to support staff in this work. Workload planning models need to incorporate curriculum development work to ensure annual updating is quality assured.
• *Flexibility to use external experts:* Administrative processes within higher education institutions need to be flexible to more easily allow external experts access to support material development and training.

## 6 The global picture

It must be acknowledged that our account is only one part of a much bigger picture. Decolonising the curriculum prepares students for the global market. However, changes in LMICs’ public health architecture is required to give voices to those who are historically marginalised and to target the power play that shapes the hierarchal constructs of inequality, diversity, inclusivity and decolonisation. These LMICs must be supported to be ready to create a whole society approach to decolonisation, including changes to public health legislation, policies and practices.

## 7 Summary

We share this account of our decolonisation of the public health curriculum as a starting point for an ongoing narrative that aims to shift the curriculum development culture. The postgraduate public health team will continue to monitor progress and challenge staff to revise and broaden the content they use in their teaching each year, gaining new input from each cohort. We will continue to explore new ways of teaching and assessing our public health content, as it is not just what is taught, but how it is learnt and taught that will build inclusivity into the curriculum.

We hope that by sharing our progress we can inspire greater momentum toward a decolonised and diversified curriculum across the international public health education community. As the student population in higher education continues to become increasingly diverse, decolonisation and diversification of the curriculum is vital in creating a fair and inclusive learning environment that considers the needs and experiences of all students on the course and fosters a ‘sense of belonging’, enabling all students to reach their academic potential.

## Declaration of conflict of interest

Dr Joanne Morling is an author on our commentary and was involved in the work for the project, and is an editor for the journal. As such, we have declared a conflict of interest for this author.

There are no other author conflicts to declare.

## Declaration of interest

Dr J Morling is an editor for the Journal. No other author conflicts to declare.
